# Simplified model of the frequency dependence of the LFP’s spatial reach

**DOI:** 10.1186/1471-2202-13-S1-P144

**Published:** 2012-07-16

**Authors:** Szymon Łęski, Henrik Lindén, Tom Tetzlaff, Klas H Pettersen, Gaute T Einevoll

**Affiliations:** 1 Department of Neurophysiology, Nencki Institute of Experimental Biology, Warsaw, 02-093, Poland; 2 CIGENE, Department of Mathematical Sciences and Technology, Norwegian University of Life Sciences, Ås, 1432, Norway; 3 Department of Computational Biology, School of Computer Science and Communication, Royal Institute of Technology (KTH), Stockholm, 10044, Sweden; 4 Institute of Neuroscience and Medicine (INM-6), Computational and Systems Neuroscience, Research Center Jülich, 52425, Germany

## 

**Figure 1:**
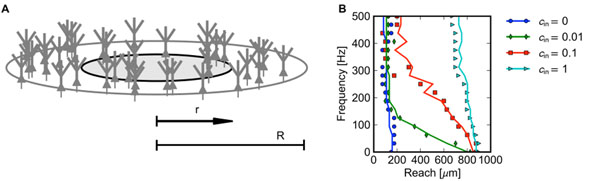
A. Model setup, *r* (reach) and *R* defined in the text. B. The reach *r* at the soma level as a function of LFP frequency and input correlation for a population of layer 5 pyramidal neurons stimulated basally, symbols – full simulation, lines – simplified model. The input correlation is defined as a an average fraction of synaptic currents shared by each pair of neurons.

One of the fundamental questions regarding the local field potential (LFP), the low-frequency part of the extracellularly recorded electric potential, is how far the signal propagates in the brain [1]. We have previously shown [2] that the low-pass filtering in dendrites [3] leads to a frequency dependent spatial spread of the LFP. These previous results were obtained by simulating a large population of morphologically reconstructed neurons. The cells were placed homogeneously within a disc of radius *R* = 1mm (Figure 1A). We defined the *reach* of the LFP as a radius *r* <*R* such that the cells located beyond that radius contributed no more than 5% of the total amplitude at the center of the population. We showed that the reach depends, among other factors, on the input correlation and the frequency.

Here we employ a simplified model of the population to identify the two main effects behin the frequency dependence of the reach: 1) frequency dependence of the ‘transition distance’, that is, the distance beyond which a single cell can be approximated as a dipole, 2) frequency dependence of the mean pairwise correlation of the single neuron contributions to the LFP. The simplified model is in agreement with the full simulation results if both effects are taken into account (Figure 1B), while neither of the factors alone is sufficient.
